# Nanobody Nb6 fused with porcine IgG Fc as the delivering tag to inhibit porcine reproductive and respiratory syndrome virus replication in porcine alveolar macrophages

**DOI:** 10.1186/s13567-020-00868-9

**Published:** 2021-02-17

**Authors:** Lu Zhang, Lizhen Wang, Shuaishuai Cao, Huanhuan Lv, Jingjing Huang, Guixi Zhang, Kaissar Tabynov, Qin Zhao, En-Min Zhou

**Affiliations:** 1 Department of Preventive Veterinary Medicine, College of Veterinary Medicine, Northwest A&F University, Yangling, 712100 Shaanxi China; 2 Scientific Observing and Experimental Station of Veterinary Pharmacology and Diagnostic Technology, Ministry of Agriculture, Yangling, 712100 Shaanxi China; 3 Kazakh National Agrarian University, 050010 Almaty, Kazakhstan

**Keywords:** nanobody, nanobody-pFc, PRRSV, permissive cell targeting, antiviral agents

## Abstract

Porcine reproductive and respiratory syndrome virus (PRRSV) is a highly contagious virus that has led to enormous economic loss worldwide because of ineffective prevention and treatment. In view of their minimized size, high target specificity and affinity, nanobodies have been extensively investigated as diagnostic tools and treatments of many diseases. Previously, a PRRSV Nsp9-specific nanobody (Nb6) was identified as a PRRSV replication inhibitor. When it was fused with cell-penetrating peptide (CPP) TAT, Nb6-TAT could enter the cells for PRRSV suppression. However, delivery of molecules by CPP lack cell specificity and have a short duration of action. PRRSV has a tropism for monocyte/macrophage lineage, which expresses high levels of Fcγ receptors. Herein, we designed a nanobody containing porcine IgG Fc (Fcγ) to inhibit PRRSV replication in PRRSV permissive cells. Fcγ fused Nb6 chimeric antibody (Nb6-pFc) was assembled into a dimer with interchain disulfide bonds and expressed in a *Pichia pastoris* system. The results show that Nb6-pFc exhibits a well-binding ability to recombinant Nsp9 or PRRSV-encoded Nsp9 and that FcγR-mediated endocytosis of Nb6-pFc into porcine alveolar macrophages (PAM) was in a dose-dependent manner. Nb6-pFc can inhibit PRRSV infection efficiently not only by binding with Nsp9 but also by upregulating proinflammatory cytokine production in PAM. Together, this study proposes the design of a porcine IgG Fc-fused nanobody that can enter PRRSV susceptible PAM via FcγR-mediated endocytosis and inhibit PRRSV replication. This research reveals that nanobody-Fcγ chimeric antibodies might be effective for the control and prevention of monocyte/macrophage lineage susceptible pathogeneses.

## Introduction

Antibodies represent the largest and fastest growing class of drugs against numerous diseases in the pharmaceutical industry. Although many conventional monoclonal antibodies (mAbs) have proven therapeutic potential in the treatment of different diseases, their preparation is usually time-consuming and costly, specifically for antibody manufacturing processes in eukaryotic systems. These disadvantages inhibit their clinical use for disease control in animals. In recent years, nanobodies (Nbs) derived from single-domain antibodies (sdAbs) of the *Camelidae* immunoglobulin have become an attractive alternative to mAbs due to their smaller size, greater solubility, easy expression by prokaryotic or eukaryotic systems, and low cost production [1–3]. More importantly, Nbs carrying different tags can enter target cells, making them particularly suitable for the treatment of the diseases [4]. While many works on the production and design of Nbs for treatment of human diseases are currently available [5], there are limited studies on the preparation of Nbs against animal diseases.

Porcine reproductive and respiratory syndrome (PRRS) mainly causes reproductive failures in sows and respiratory distress in growing pigs [6, 7]. The disease has been considered an epidemic for more than 30 years [8–11] and remains a great concern to the swine industry worldwide due to viral mutation, ineffective vaccinations, and lack of efficient antiviral strategies [12–14]. PRRS virus (PRRSV), the causative agent of PRRS, is a single stranded positive RNA virus that belongs to the *Arteriviridae* family [15, 16]. PRRSV isolates can be divided into two genotypes: European (Type 1) and North American (Type 2). The complete genome of PRRSV varies from 14.9 to 15.5 kb in length and contains 11 open reading frames (ORF) [17, 18]. Amongst these, ORF1b encodes the Nsp9 gene, the RNA-dependent RNA polymerase (RdRp), which is denoted as one of the core factors in PRRSV replication. Previous studies documented that Nsp9 is a relatively conserved region in the PRRSV genome [19] and an ideal target of antiviral design for the control of PRRSV infection. In our previous study, we produced Nb6 fused with trans-activating transduction (TAT) peptide and found that it can inhibit replication of two genotypes of PRRSV isolates in MARC-145 cells and PAM [10, 20]. However, TAT exhibits several disadvantages including a short half-life in blood and nonspecific delivery to normal tissue [21].

As it is well known, PAM serve as the primary host cells for PRRSV infection and express several phagocytic receptors that play a role in macrophage receptor-mediated phagocytosis [22]. The most effective phagocytic leukocyte receptors have been reported as the receptors for the Fc portion of IgG (FcγRs) [23, 24]. Activation of FcγRs can secrete cytokines by triggering various secondary signaling pathways and produce a variety of cytokines, chemokines, and lipid mediators [25, 26]. On the basis of the signaling motifs at their intracellular domain, type I FcγRs are divided into activation and inhibitory receptors [27]. So far, three types of porcine FcγRs (FcγRI, FcγRIII and FcγRIIB) have been confirmed on PAM, including two activation receptors (FcγRI and FcγRIII) and one inhibitory receptor (FcγRIIB) [28–33]. Importantly, after the Fc portion of IgG binds to activated FcγRs, the immunoreceptor tyrosine based activation motif (ITAM) domains become phosphorylated triggering a series of signals to mediate particle internalization [34].

In this study, a pig Fcγ ligated PRRSV Nsp9 specific nanobody (named Nb6-pFc) was designed, produced and shown to inhibit PRRSV infection of PAM. Using Western blot, immunofluorescence assay (IFA), and flow cytometry assay (FCM), the uptake of Nb6-pFc by PAM via FcγR-mediated phagocytosis and subsequent inhibition of PRRSV replication were evaluated. Overall, this study provides a novel approach for the development of nanobodies fused with IgG Fc as therapeutic agents to control PRRSV infection.

## Materials and methods

### Cells and viruses

PAM were collected from 6-week-old PRRSV-negative pigs using the method previously described [35]. Cells were cultured in RPMI-1640 medium (Gibco, USA) containing 10% heated-inactivated fetal bovine serum (FBS) and an antibiotic–antimycotic solution (100 μg/mL of streptomycin, 100 μg/mL of penicillin) at 37 °C in a humidified 5% CO_2_ incubator. MARC-145 cells were cultured in DMEM medium (Gibco, USA) containing 10% FBS. In this study, a green fluorescent-labeled recombinant PRRSV named rSD16/TRS2/clover [36] and a highly pathogenic PRRSV (HP-PRRSV)GD-HD (GenBank ID: KP793736.1) were selected for virus inhibition experiments. All PRRSV strains were propagated and titrated in MARC-145 cells and stored at − 80 °C.

### Design and production of nanobodies and pig IgG Fc fusion proteins

**Figure 1:**
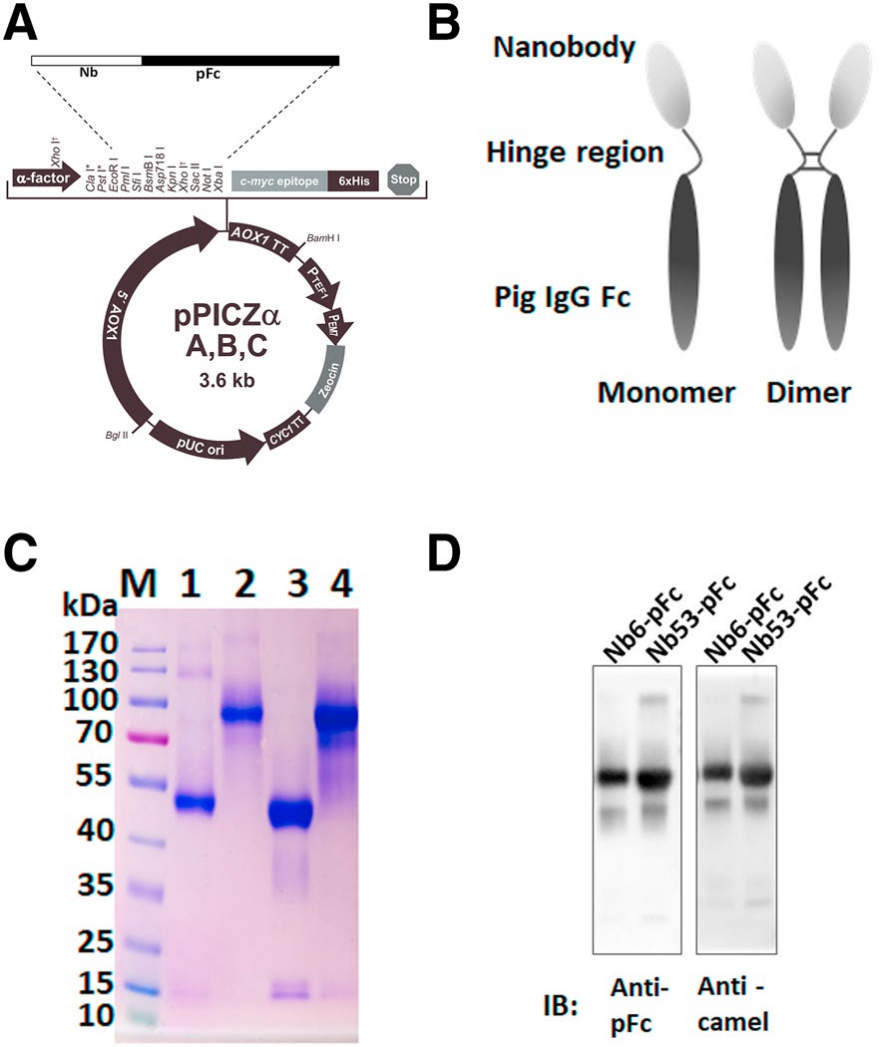
**Construction and analysis of purified pig Fc linked nanobodies.** **A** Design and construction of pPICZα-A-Nbs-pFc. **B** Speculative structural model of Nbs-pFc. **C** SDS-PAGE identification of purified Nbs-pFc. The predicted sizes of the pFc linked nanobodies are 80 kDa in absence of β-mercaptoethanol and 40 kDa in presence of β-mercaptoethanol. M, protein marker; lanes 1 to 4, reduced Nb6-pFc, non-reduced Nb6-pFc, reduced Nb53-pFc, non-reduced Nb53-pFc, respectively. **D** Western blot identification of purified pig Fc-linked nanobodies by HRP-conjugated goat anti-swine IgG and rabbit anti-camel sera.

To obtain pig Fcγ fused nanobodies (named Nbs-pFc), the gene of pFc (including hinge region, CH2, CH3) was ligated downstream of the Nb gene. The sequences of Nb6 and Nb53 (a nanobody derived from unimmunized camel peripheral blood lymphocytes, as the control) were obtained from previous research [10]. The template of porcine IgG1a Fc (accession number U03781) was retrieved from *The International Immunogenetics Information System* (IMGT) website. The full-length Nb6-pFc and Nb53-pFc strands were codon-optimized for yeast and synthesized by GENEWIZ (China). Then, restriction endonucleases EcoRI and XbaI were added to Nb6-pFc and Nb53-pFc by the primers shown in Table 1, which were then inserted into the *Pichia pastoris* expression vector pPICZαA (Invitrogen, USA) (Figure 1A). The reverse primer contains a stop codon to terminate the expression of the following sequences in vectors (Table 1). Based on Figure 1B, the Nbs-pFc protein was determined to be a dimer with an interchain disulfide bond. After single digestion by SacI (TakaRa, China), the linearized pPICAαA-Nb6-pFc and pPICAαA-Nb53-pFc were electro-transformed into a X-33 *Pichia pastoris* strain and placed in YPD (containing 1% yeast extract, 2% peptone, and 2% dextrose) plates with 100 μg/mL Zeocin™ (Gibco, USA). Plasmids with positive X-33 colonies were identified by PCR using the Nbs-pFc primer pair (Table 1). After the recombinant strains were activated in YPD medium for 2 days, their cultivation was enlarged in BMGY medium (containing 1% yeast extract, 2% peptone, 100 mM potassium phosphate, 1.34% YNB, 4 × 10^–5^% biotin, and 1% glycerol) for 1 day, and then induced by 0.5% methanol for expression in BMMY (containing 1% yeast extract, 2% peptone, 100 mM potassium phosphate, 1.34% YNB, 4 × 10^–5^% biotin, and 0.5% methanol). Following 5 days of induction, the culture medium of *Pichia pastoris* was collected, and pH was adjusted to 7.5 using 1 M Tris-Base for purification by Protein G resin (Genscript, China). All reagents were purchased from Sigma, Germany, and the procedures for expression were performed according to the Easyselect™ *Pichia* Expression Kit (Invitrogen, USA) manual.

**Table 1:** Primers for construction of pPICZα A-Nbs-pFc plasmids and detection of PRRSV ORF7 and cytokines

Gene	Primer	Sequence (5′–3′)
Nbs-pFc	Forward	GGTGAATTCCAGGTCCAACTGCAGGAGTC
	Reverse	GGGTCTAGA*TCAC*TTGCCCTGTGTCTTGC
PRRSV ORF7	Forward	AGATCATCGCCCAACAAAAC
	Reverse	GACACAATTGCCGCTCACTA
PAM IL-1β	Forward	CCTTGAAACGTGCAATGATGACT
	Reverse	GTGGAGAGCCTTCAGCATGT
PAM IL-6	Forward	CCGGTCTTGTGGAGTTTCAG
	Reverse	CAGGGTCTGGATCAGTGCTT
PAM IL-8	Forward	CCAGCATTCACAAGTCTTCTTGC
	Reverse	ATGTCCTCAAGGTAGGATGGGG
PAM IL-10	Forward	GCATCCACTTCCCAACCA
	Reverse	TCGGCATTACGTCTTCCAG
PAM IL-12p40	Forward	GTTTCAGACCCGACGAACTC
	Reverse	GAGGACCACCATTTCTCCAG
PAM IFN-α	Forward	ACTTCCACAGACTCACCCTCTATC
	Reverse	ATGACTTCTGCCCTGATGATCT
PAM IFN-β	Forward	TGCAACCACCACAATTCC
	Reverse	CTGAGAATGCCGAAGATCTG
PAM TNF-α	Forward	AGCCGCATCGCCGTCTCCTAC
	Reverse	CCTGCCCAGATTCAGCAAAGTCC
GAPDH	Forward	CCTTCCGTGTCCCTACTGCCAAC
	Reverse	GACGCCTGCTTCACCACCTTCT

Underlined and italicized nucleotides sequences are the restriction enzyme cutting sites nucleotides and the termination codon, respectively

### Indirect enzyme-linked immunosorbent assay (ELISA)

To determine Nb6-pFc binding with Nsp9, a modified ELISA was designed and performed as previously described [10]. The recombinant Nsp9 was diluted with 0.01 M phosphate-buffered saline (PBS) and coated into an ELISA plate (Nunc, Denmark) at 400 ng/well overnight at 4 °C. After washing with PBS’T (5% of Tween-20 in 0.01 M PBS) three times, the plates were blocked with 200 μL of 2.5% dry milk (BD, USA) in PBS’T for 1 h at room temperature. Then, different dilutions of Nb6-pFc, Nb53-pFc, and His-Nb6 (stored from earlier studies) [20] were added to the plate as the first antibody to incubate for 1 h at 37 °C. After washing again, the rabbit anti-camel IgG sera (1:2000) were added and incubated for 1 h at 37 °C. Then, the peroxidase-goat anti-rabbit IgG (H+L) (Jackson, USA) were added and incubated for 1 h at 37 °C. After a second washing, the single-component substrate solution tetramethylbenzidine (TMB) (Solarbio, China) was added to produce a color reaction. Finally, 3 M H_2_SO_4_ was used to stop the reaction, and OD values were read at 450 nm (OD_450 nm_) by an automated ELISA plate reader.

### Pull-down assay

To determine whether Nb6-pFc interacts with Nsp9 from PRRSV, a pull-down assay was designed and performed. Briefly, MARC-145 cells were inoculated with PRRSV strain GD-HD at a multiplicity of infection (MOI) of 0.1 for 36 h. Then, the cells were lysed with NP40 (Beyotime, China) containing a proteinase inhibitor cocktail (Roche, Mannheim, Germany), and the insoluble material was removed by centrifugation at 13 000 × *g* for 10 min at 4 °C. The Nb6-pFc and control antibody were first incubated with Dynabeads protein G (Invitrogen, USA) following the manufacturer’s instructions, then mixed with the above-mentioned cell lysate and incubated overnight at 4 °C. Magnetic beads and protein complex were separated from the liquid by a magnet, washed with PBS three times, then analyzed for Western blot analysis.

### Analysis of Nbs-pFc uptake in PAM

PAM were seeded in 24-well plates at a density of 1 × 10^6^/mL. After confluent monolayers formed, the cells were incubated with 3% FBS culture medium containing Nb6-pFc, Nb53-pFc, and His-Nb6 at concentrations of 5–20 μM at 4 °C for 1 h. Then, cells were shifted into a 5% CO_2_ humidified incubator at 37 °C. After a set period of time (4 °C for 1 h; 37 °C for 5, 15, 30 min and 1, 2, 6, 12, 24 h), the cells were collected, washed with PBS three times, and detected by Western blot, IFA, and FCM.

### Cell viability analysis

To evaluate the cytotoxicity of Nbs-pFc, cell viability was evaluated using a cell counting kit-8 (CCK-8) assay (Beyotime, China) as described previously [35] with the following modifications. PAM were seeded at a density of 1 × 10^5^ cells per well in 96-well plates and incubated in a 5% CO_2_ humidified incubator at 37 °C. After confluent monolayers formed, the cells were washed with PBS and then incubated with different concentrations of Nb6-pFc and Nb53-pFc in 3% FBS medium for 24 h. CCK-8 reagent (10 μL) was added into each well and incubated for 1 h at 37 °C. The absorbance was measured at OD_450 nm_ using an Epoch microplate spectrophotometer (BioTek Instruments, Winooski, VT, USA) to calculate cell viability. The results are expressed as the percentage of the optical density of treated cells to that of untreated control cells, which was defined as 100% viability.

### Indirect immunofluorescence assay (IFA)

The IFA procedure was modified based on a previously reported assay [37]. The cells were fixed with 4% paraformaldehyde (Sigma-Aldrich) for 15 min at room temperature, and then permeated with 0.25% Triton X-100 (Sigma-Aldrich) for 5 min. After washing with PBS three times, the fixed cells were blocked by 1% BSA for 30 min at room temperature. After three more washes with 0.01 M PBS, Nbs-pFc (10, 1, 0.1 μg/mL), mouse anti-Nsp9 sera (homemade, 1:1000 dilution) or rabbit anti-camel sera (homemade, 1:1000 dilution) were incubated with cells as the first antibody. After a second washing, Alexa flour 488 conjugated-goat anti-swine IgG (H+L) (Jackson, USA), Alexa flour 555 conjugated-goat anti-mouse IgG (H+L) (Thermo Fisher Scientific, USA), or Alexa flour 488 conjugated-goat anti-rabbit IgG (H+L) (Jackson, USA) were used as the secondary antibodies. Following another washing, the coverslips were dried and mounted on microscope slides using fluoroshield with DAPI (Sigma, Germany) and observed under a confocal microscope (AF6000; Leica, Wetzlar, Germany). Mock-inoculated or medium-treated cells were used as controls to assess background staining.

### Flow cytometry assay (FCM)

FCM was performed following the procedure described by the manufacturer (Invitrogen, CA, USA). First, PAM were seeded in 24-well plates at a density of 1 × 10^6^/mL. After 2 h of incubation with Nbs-pFc, the cells were released from cell plates by trypsin digestion (Sigma, Germany). After washing with PBS, the cells were fixed and permeablized by a 1-mL fixation/permeabilization working solution in Foxp3/Transcription Factor Staining Buffer Set (Thermo Fisher Scientific, USA) at 4 °C for 40 min in a 1.5-mL tube. Then the cells were blocked by 1% BSA for 1 h at room temperate and then incubated with Alexa Flour 488-conjugated goat anti-swine IgG (1:200, Jackson, USA) for 1 h on ice. Finally, the cells were resuspended in a flow cytometry staining buffer and analyzed by flow cytometry.

### Inhibition of PRRSV replication by Nb6-pFc

PAM were seeded into 96-well plates or 12-well plates at a density of 1 × 10^6^ cells/mL and inoculated with PRRSV at a MOI of 0.01 for 1 h. Then, the cell culture medium was replaced with fresh medium supplemented with Nb6-pFc, Nb53-pFc, or purified specific pathogen-free pig IgG. At 24 and 36 h post-inoculation (hpi), for SD16/TRS2/clover inoculated cells, virus fluorescence was observed by an inverted microscope (Leica DMI6000B, Leica, Germany); for GD-HD inoculated cells, cell supernatant were collected for the progeny virus titration, and the cells were harvested for Western blot and quantitative real-time PCR analyses.

### Quantitative real-time PCR

The PAM were washed three times with PBS, and the total RNA were extracted using TRIzol reagent (TaKaRa, China). Reverse transcription reactions were performed using a PrimeScript RT master mix kit (TaKaRa, China) according to the manufacturer’s instructions. Quantitative real-time (qPCR) was carried out on a StepOnePlus real-time PCR system (Applied Biosystems, Foster City, CA, USA) using RealStar green fast mixture with ROX (Roche, Germany) and PRRSV ORF7, IL-1β, IL-6, IL-8, IL-10, IL12p40, IFN-β, IFN-α, and TNF-α forward and reverse primer pairs (list in Table 1). The reaction was performed in 20 μL, and cellular glyceraldehyde-3-phosphate dehydrogenase (GAPDH) mRNA was measured as an internal reference.

### Virus titration

Progeny virus production was detected by titration as previously described [38], with the following modifications. MARC-145 cells were plated on 96-well plates 24 h before viral inoculation. Virus supernatants were tenfold serially diluted, and 100 μL of each was added to each well with eight replicates. Five days after inoculation, the 50% tissue culture infective dose (TCID_50_) was calculated using the Reed–Muench method [39].

### Western blot analysis

Western blot was performed as previously described [40], with the following modifications. Briefly, cells were harvested and lysed, and the cellular proteins were separated by 12% SDS-PAGE and blotted onto polyvinylidene difluoride (PVDF) membranes (Millipore, USA). After being blocked with 5% skim milk for 1 h at room temperature, the PVDF membranes were probed with one of the following primary antibodies: rabbit anti-camel IgG serum (1:2000), mouse anti-PRRSV N mAb 6D10 (1 μg/mL; produced in our laboratory), or anti-β-tubulin (1:5000; Transgene, Beijing, China). Membranes were washed three times with PBST, followed by incubation with HRP-conjugated goat anti-rabbit IgG (Jackson, USA) or HRP-conjugated goat anti-mouse IgG (Jackson, USA) at a 1:5000 dilution as the secondary antibody. The reactions were visualized using an ECL chemiluminescent detection system according to the manufacturer’s instructions (Pierce, Rockford, IL, USA).

### Statistical analysis

All experiments were performed with at least three independent replicates. The results were analyzed using a student t test for comparisons between two groups or one-way analysis of variance (ANOVA) for more than two groups. A *P* value of 0.05 was considered statistically significant.

## Results

### Expression and purification of nanobodies fused with pig IgG Fc

The nanobodies fused to pig IgG Fc, denoted Nb6-pFc and Nb53-pFc, were successfully expressed and secreted in the culture medium from *Pichia pastoris*. The two fusion proteins were affinity purified using Protein G resin (Genscript, China), then analyzed by SDS-PAGE (reduced and unreduced) (Figure 1C) and Western blot (Figure 1D). The results reveal that high purity Nb6-pFc and Nb53-pFc were obtained. In the presence of β-mercaptoethanol, the predicted size of Nbs-pFc was about 40 kDa, while unreduced SDS-PAGE (without β-mercaptoethanol) exhibited a size 2-times larger (about 80 kDa). These results suggest that Nbs-pFc can be assembled into a dimer by interchain disulfide bonds located in the hinge region (amino acid sequences: GTKTKPP*C*PI*C*P) [41], which is consistent with the speculative structural model (Figure 1B). Purified Nbs-pFc could be detected by rabbit anti-camel serum and HRP-conjugated goat anti-swine IgG (Figure 1D), indicating that they contained the Nb-pFc sequences. Collectively, these findings show that Nb6-pFc and Nb53-pFc (as the control) were successfully prepared.

### Interaction between Nb6-pFc and PRRSV Nsp9

**Figure 2:**
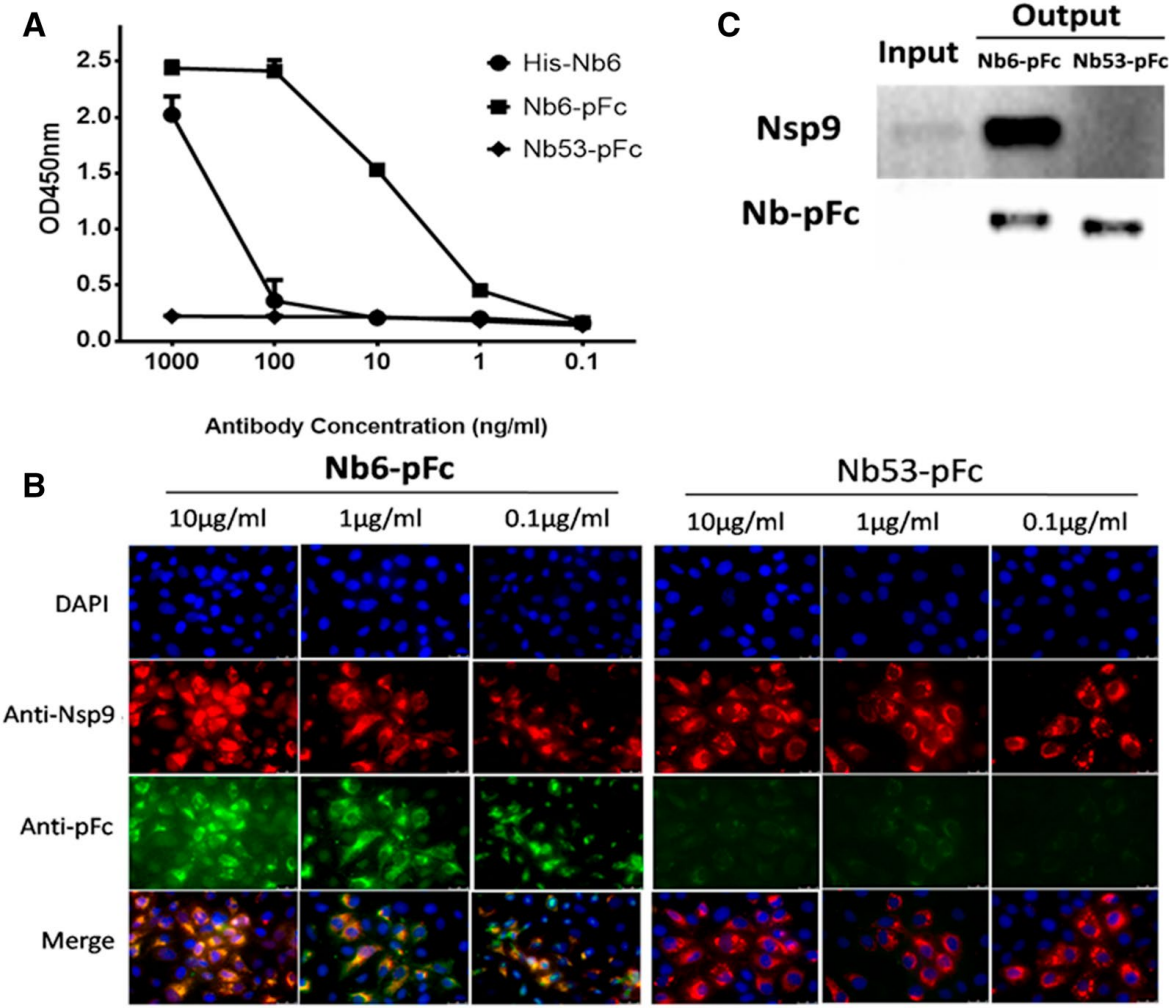
**Detection of Nb6-pFc interacted with Nsp9.** **A** ELISA detection of binding with prokaryotic expressedNsp9. Different concentrations of Nb6-pFc interacted with Nsp9. His-Nb6 and Nb53-pFc were used as positive and negative antibody controls, respectively. Assays were performed in duplicate, and data are presented as mean ± SD. **B** IFA detection of binding with PRRSV Nsp9. Nb6-pFc and Nb53-pFc were detected by Alexa flour 488-conjugated goat anti-swine IgG (Green) and positive infected cells were stained by mouse anti-Nsp9 serum (Red). **C** MARC-145 cells were inoculated with SD16 at a MOI of 0.1 for 36 h, and Nsp9 was pulled down by Nb6-pFc and Nb53-pFc. The bound proteins were detected by Western blot using mouse anti-Nsp9 serum and HRP-conjugated goat anti-swine IgG Fc.

Indirect ELISA was used to evaluate the Nb6-pFc binding with Nsp9. As shown in Figure 2A, Nb6-pFc and His-Nb6 specifically reacted with Nsp9 produced by bacterial expression systems, but Nb53-pFc did not. The binding concentration of Nb6-pFc to Nsp9 was as low as 1 ng/mL (Figure 2A) indicating that Nb6-pFc produced by the *Pichia pastoris* expression system has better antigen binding activity.

In order to verify the binding of Nb6-pFc to PRRSV Nsp9, MARC-145 cells were inoculated by PRRSV GD-HD strains at an MOI of 0.1 for 24 h. Then, the cells were fixed and detected by Nb6-pFc or Nb53-pFc and homemade mouse anti-Nsp9 sera. As shown in Figure 2B, 10, 1.0 and 0.1 μg/mL Nb6-pFc displayed positive green fluorescence of PRRSV infected cells and colocalization with mouse anti-Nsp9 positive cells (red), while each concentration of Nb53-pFc displayed no fluorescence (Figure 2B). Subsequently, the PRRSV Nsp9 in infected MARC-145 cells was also pulled down by the Nb6-pFc but not by Nb53-pFc (Figure 2C). These results indicate that Nb6-pFc expressed by *Pichia pastoris* can interact with PRRSV Nsp9.

### Cellular uptake of Nb6-pFc and Nb53-pFc

**Figure 3:**
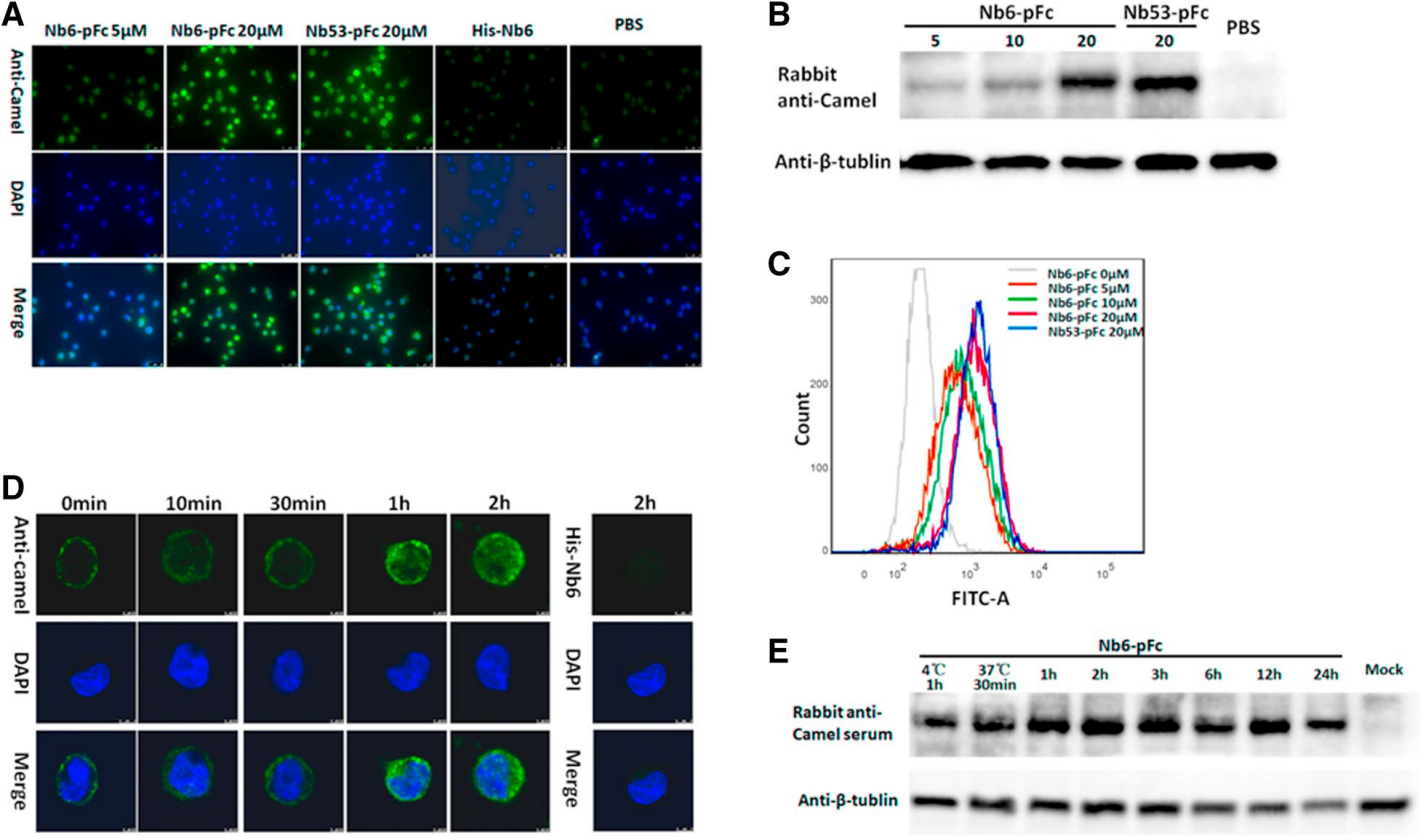
**Cellular uptake of Nbs-pFc by PAM.** **A** IFA, **B** Western blot, and **C** flow cytometry analyses of Nbs-pFc entry into PAM at different concentrations. PAM were treated with 0–20 μM Nbs-pFc for 2 h. **D** IFA and **E** Western blot analyses of uptake of 20 μM Nb6-pFc into PAM at different time points of incubation.

**Figure 4:**
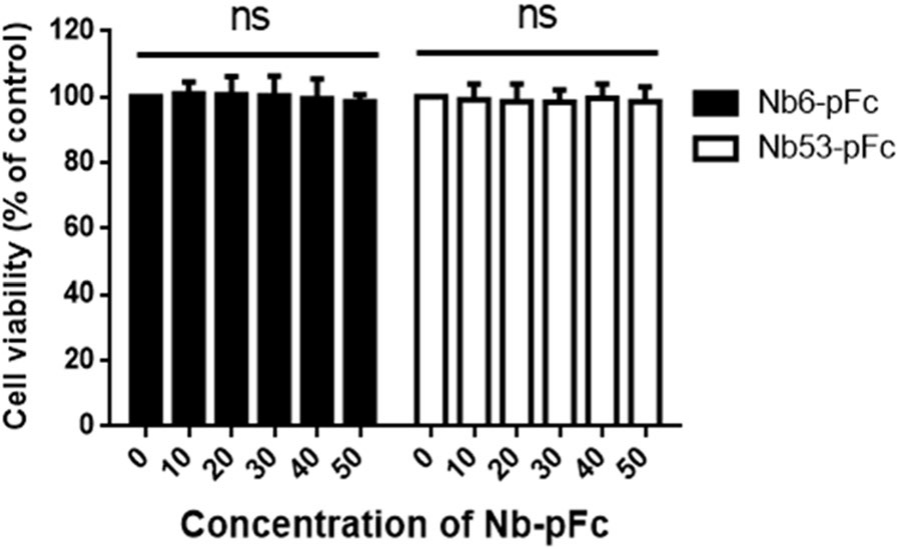
**Detection of Nbs-pFc toxicity using CCK8 kits.** Data are expressed as mean ± SD from five independent experiments. *P* values were calculated using ANOVA. *P* values were < 0.05 (*), < 0.01 (**), and < 0.001 (***) compared with normal cultured cells (ns: not significant).

To examine the ability of Nbs-pFc to enter PAM, cells treated with Nbs-pFc were detected by Western blot, IFA and FCM. According to IFA results (Figure 3A), Nb6-pFc and Nb53-pFc were clearly detected in PAM at concentrations of 5 and 20 μM, respectively; in contrast, His-Nb6 without pFc was not detected in the cells. These results were also verified by Western blot (Figure 3B), confirming that Nb6-pFc accumulated in the cytoplasm in different concentrations. FCM analysis indicates that Nb6-pFc internalization was dose-dependent in PAM (Figure 3C). The delivery efficiency of the control nanobody Nb53-pFc at 20 μM was similar to that of Nb6-pFc when detected by Western blot and FCM (Figures 3B, C). In addition, the time points at which Nb6-pFc entered PAM were determined by IFA (Figure 3D) and Western blot (Figure 3E). After 1 h of treatment at 4 °C, Nb6-pFc was clearly detected on the cytomembrane, and then gradually entered the cytoplasm within 2 h after shifting to 37 °C (Figures 3D, E); the presence of Nb6-pFc persisted in the cells up to 24 h (Figure 3E). Furthermore, a cell counting kit-8 (CCK-8) assay was performed to test the toxicity of Nb6-pFc in PAM. As shown in Figure 4, the viability of PAM was close to that of the untreated control cells until a concentration of 50 μM Nb6-pFc was added, suggesting that Nb6-pFc is non-toxic to PAM at lower concentrations. Moreover, Nbs-pFc can be uptaken by PAM in a dose-dependent manner because of interaction between porcine IgG Fc and FcγRs. It should also be noted that porcine IgG1 Fc chimeric nanobodies displayed no cell toxicity.

### Nb6-pFc inhibits PRRSV replication in PAM

To evaluate the antiviral efficacy of Nb6-pFc, a clover-tagged PRRSV infectious clone and HP-PRRSV strain GD-HD were selected for subsequent testing.

**Figure 5:**
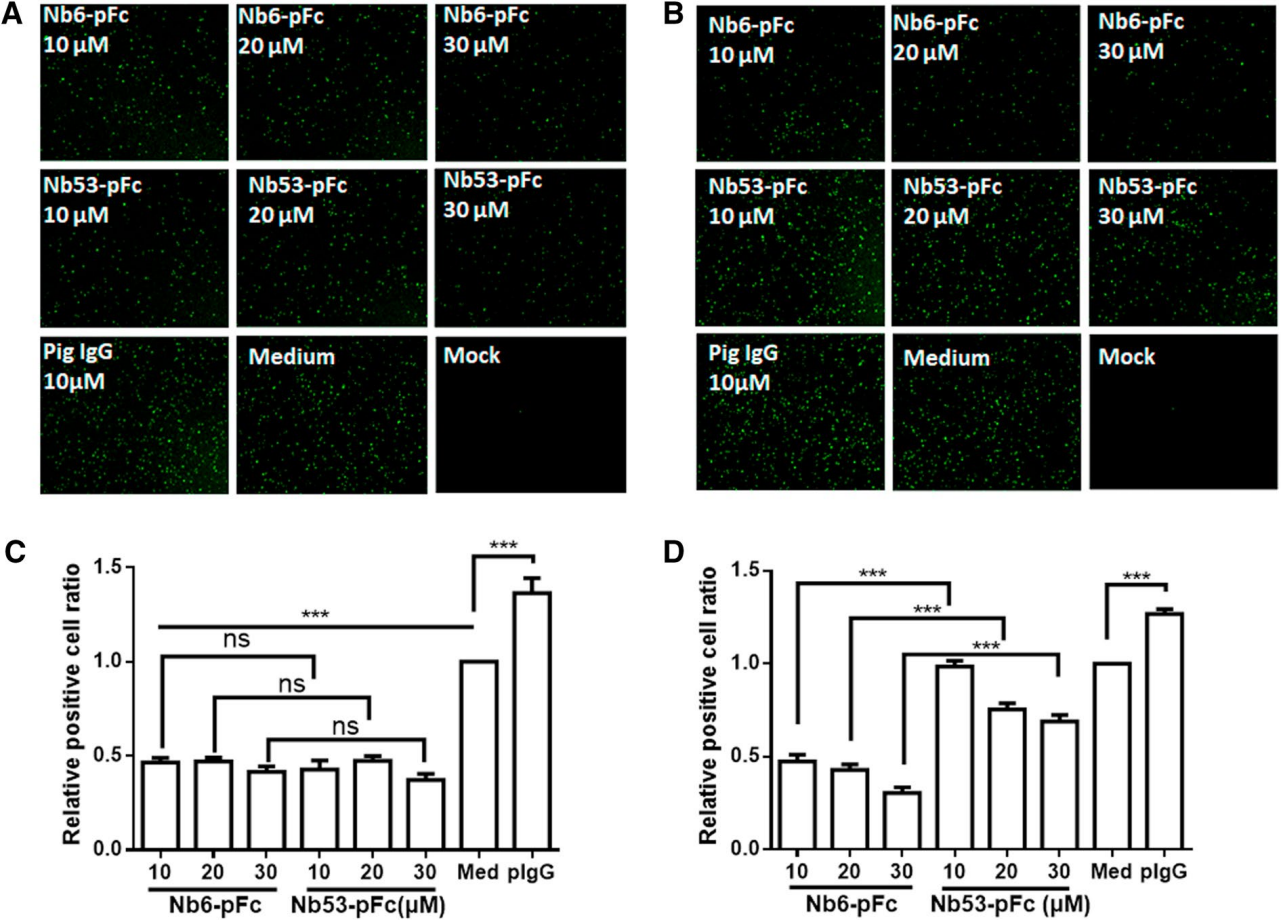
**Inhibition of rSD16/TRS2/clover replication by Nb6-pFc in PAM.** Fluorescence was observed with a microscope (Leica DMI6000B Leica, Germany) at **A** 24 hpi and **B** 36 hpi, and PRRSV positive particles were calculated using Image J software and compared with the medium control group to estimate inhibition efficiency of Nb6-pFc at **C** 24 hpi and **D** 36 hpi. Data are expressed as mean ± SD from three independent tests. *P* values were calculated using ANOVA as < 0.05 (*), < 0.01 (**), and < 0.001 (***) compared with cells infected with PRRSV alone (ns: not significant).

Clover-tagged PRRSV infectious clone, rSD16/TRS2/clover, offers a fast and reliable testing method for screening new antiviral agents [36] and was used to evaluate the inhibition efficiency of Nb6-pFc. The monolayer PAM were inoculated with rSD16/TRS2/clover at a MOI of 0.01 for 1 h, and were treated with 3% FBS/RIPM 1640 containing different concentrations of Nb6-pFc, Nb53-pFc, and purified pig IgG. The treated cells were cultured in an incubator for 36 h and observed by an inverted microscope (Leica DMI6000B, Leica, Germany) at 24 and 36 hpi to evaluate the replication suppression. The PRRSV positive particles were calculated using Image J software and compared with the medium control group to estimate the inhibition efficiency of Nb6-pFc (Figure 5C, D). At the time point of 24 hpi, Nb6-pFc and Nb53-pFc (with concentrations of 10, 20, and 30 μM) treated cells showed less fluorescence than 10 μM pig IgG and medium control (Figure 5A). The relative positive cell ratio suggests that each concentration of Nb6-pFc and Nb53-pFc inhibits about 50% of virus replication. Comparatively, at 36 hpi, the inhibition level of Nb6-pFc is much higher than that at Nb53-pFc in every concentration as well as pig IgG and medium control (Figure 5B). According to the calculations, 10, 20, and 30 μM Nb6-pFc showed about 50%, 45%, and 25% relative positive cell ratios compared to the medium control, respectively; the same concentrations of Nb53-pFc showed about 100%, 80%, and 75%, which were significantly higher than Nb6-pFc (Figure 5C). This further signifies that Nb6-pFc can inhibit rSD16/TRS2/clover replication in a dose-dependent manner.

**Figure 6:**
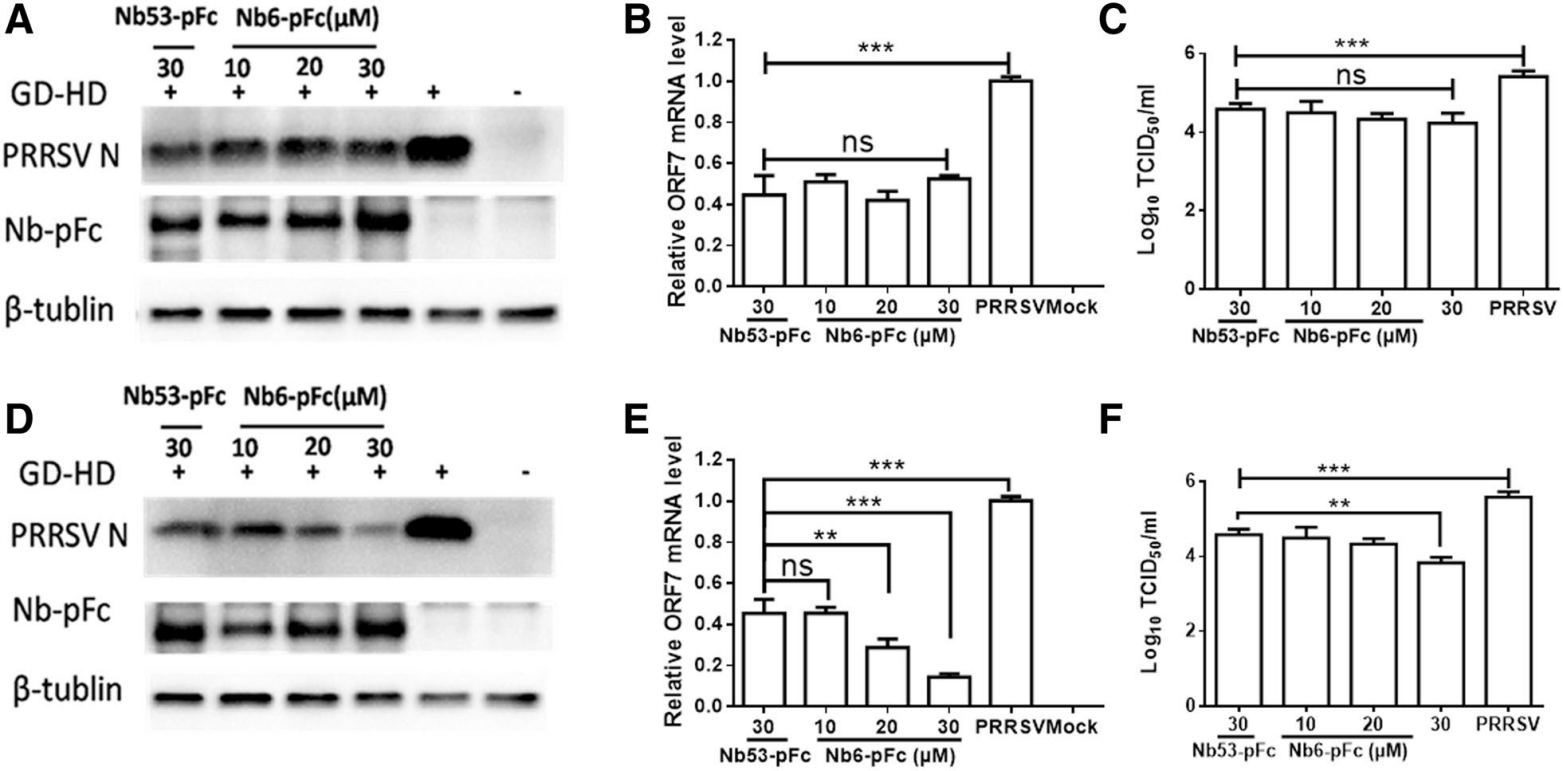
**HP-PRRSV GD-HD replication by Nb6-pFc in PAM.** Nbs-pFc and PRRSV Nsp9 were detected at **A** 24 hpi and **D** 36 hpi by Western blot using HRP-goat anti-swine IgG and anti-PRRSV N protein mAb, respectively. Relative levels of PRRSV RNA at **B** 24 hpi and **E** 36 hpi detected by RT-qPCR using PRRSV ORF7-specific primers. The GAPDH mRNA level served as an internal reference. Progeny virus released in the cell medium was measured by TCID_50_ at **C** 24 hpi and **F** 36 hpi. Data are expressed as means from three independent experiments. *P* values were calculated using ANOVA as < 0.05 (*), < 0.01 (**), and < 0.001 (***) compared with cells infected with PRRSV alone (ns: not significant).

To verify the virus suppression activity of Nb6-pFc, a high pathogenicity PRRSV strain named GD-HD was chosen for further experiments. PAM were first inoculated with GD-HD at an MOI of 0.01 for 1 h, then cell supernatants were replaced with Nb6-pFc and Nb53-pFc. Cells and culture supernatants were harvested at 24 and 36 hpi for detection of PRRSV N protein expression via Western blot, ORF7 mRNA level via qPCR, and progeny virus. At 24 hpi, N proteins (Figure 6A) and relative mRNA levels of PRRSV ORF7 (Figure 6B) and progeny virus in culture supernatants (Figure 6C) were significantly decreased following treatment with Nb6-pFc (10, 20, and 30 μM) and Nb53-pFc (30 μM) compared to the medium control (Figure 6A). No difference was observed between the various concentrations of Nb6-pFc and Nb53-pFc. These results are consistent with clover tagged SD16 (Figure 5). At 36 hpi, in the 20 and 30 μM Nb6-pFc treated group, the expression and transcription levels of PRRSV ORF7 were significantly lower than those following treatment with 30 μM Nb53-pFc (Figures 6D, E). The virus titer of 30 μM Nb6-pFc was also significantly lower than other groups (Figure 6F), suggesting that Nb6-pFc presents an effective inhibition of GD-HD production compared with control antibody Nb53-pFc at 36 hpi. Furthermore, different concentrations of Nb6-pFc and Nb53-pFc showed the same virus suppression efficiency, which was greater than the medium control group at 24 hpi regardless of PRRSV strain (Figures 5A and 6A, B, D). These results were in conflict with those of our previous studies [10, 20].

### FcγR mediated phagocytosis of Nbs-pFc induces PAM activation

**Figure 7:**
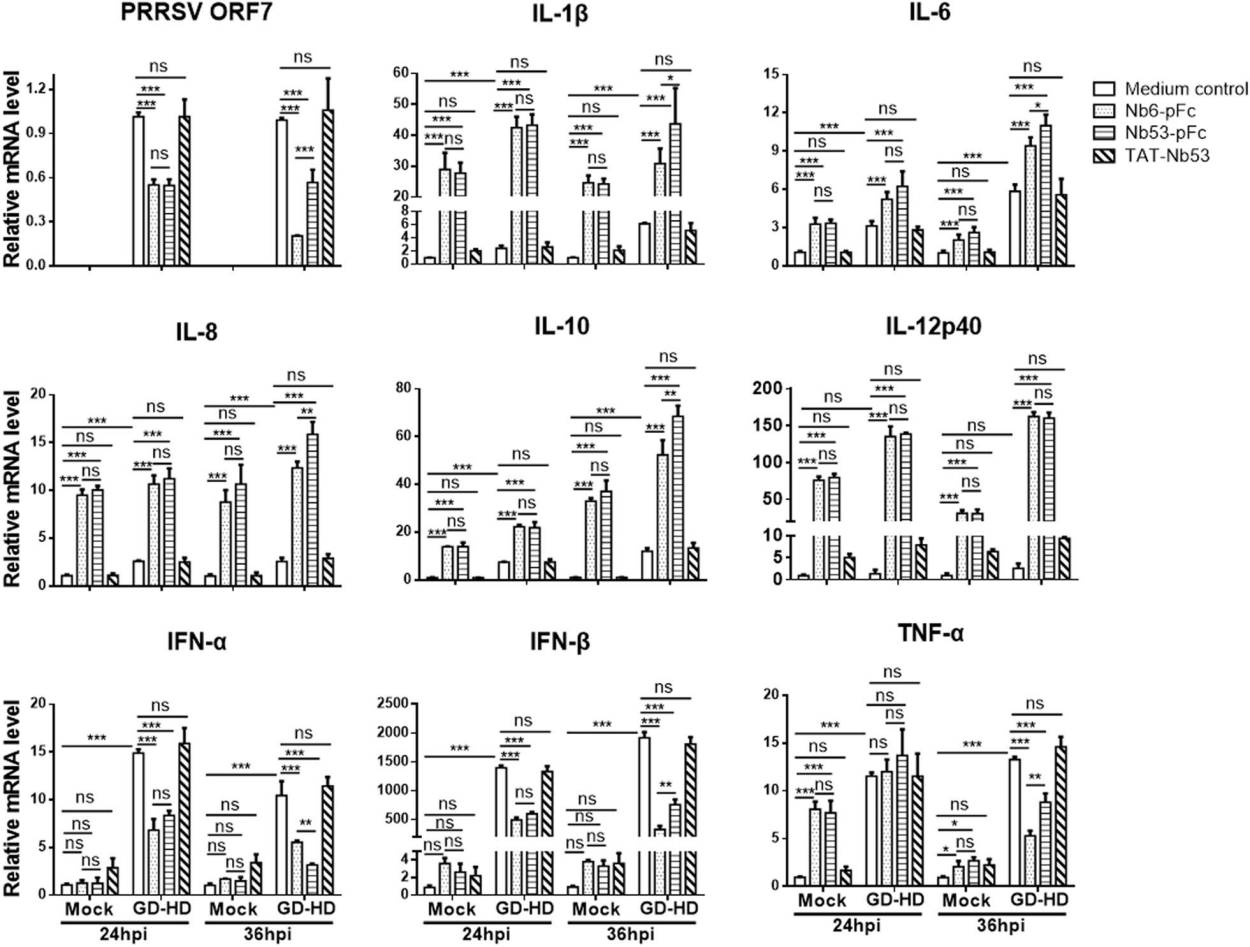
**Regulation of cytokines by Nbs-pFc with or without PRRSV infection.** PAM were innoculated with or without GD-HD (MOI of 0.01) for 1 h then treated with 30 μM of Nb6-pFc, Nb53-pFc and TAT-Nb53, and collected at 24 and 36 hpi. Relative mRNA levels of PRRSV ORF7, IL-1β, IL-6, IL-8, IL-10, IL-12p40, IFN-α, IFN-β, and TNF-α were assessed using quantitative real-time qRT-PCR. Values were normalized to the internal GAPDH control. Data are representative of three independent experiments performed in triplicate and are shown as the mean ± SD. *P* values were calculated using ANOVA as < 0.05 (*), < 0.01 (**), and < 0.001 (***) compared with cells infected with PRRSV alone (ns: not significant).

Interestingly, the findings of the current work (Figures 5A, C and 6A–C) differ from our previous research, which stated that cellular-expressed Nb53 or TAT-Nb53 do not suppress PRRSV infection [10, 20]. Considering that PAM are equipped with a broad range of receptors and can secrete cytokines by triggering various secondary signaling pathways [42], we speculated that antiviral cytokines were induced by FcγR cross-linking and phagocytosis. To prove this, PAM were inoculated with GD-HD (0.01 MOI) for 1 h, then treated with 30 μM of Nb6-pFc, Nb53-pFc or TAT-Nb53. Relative mRNA levels of PRRSV ORF7, IL-1β, IL-6, IL-8, IL-10, IL12p40, IFN-β, and TNF-α were detected by qPCR at 24 hpi and 36 hpi. As shown in Figure 7, the level of PRRSV ORF7 mRNA decreased by about 50% for both Nb6-pFc and Nb53-pFc treated cells at 24 hpi, and further decreased by 80% only for Nb6-pFc treated cells at 36 hpi, which was consistent with the results from Figures 5 and 6. PRRSV ORF7 mRNA levels were not changed from TAT-Nb53 treated cells in comparison to medium-treated cells (Figure 7). In both PRRSV inoculated and non-inoculated cells, uptake of Nbs-pFc, but not TAT-Nb53, induced dramatic changes in the cytokine production by PAM. In non-inoculated cells, after 24-h treatment with Nb6-pFc and Nb53-pFc, levels of IL-1β, IL-6, IL-8, IL-10, IL12p40, and TNF-α mRNA were elevated at least 28-fold, threefold, ninefold, 13-fold, 75-fold, and sevenfold respectively, compared with mock cells. However, IFN-α and IFN-β mRNA levels only show a slight increase, but not enough to be considered significant. Further, 36-h treatment of PRRSV non-inoculated PAM dispalyed the same trend with 24 h-treatment. At 24 and 36 hpi, PAM inoculated with 0.01 MOI of GD-HD can induce IL-1β, IL-6, IL-8, IL-10, IFN-α, IFN-β, and TNF-α expression, but not IL12p-40. After cells were treated with Nb6-pFc and Nb53-pFc, there was a larger production of cytokines than in cells inoculated with GD-HD, including IL-1β, IL-6, IL-8, IL-10, and IL-12p40 expression but not IFN-α, IFN-β, or TNF-α. The lower levels of IFN-α, IFN-β and TNF-α mRNA of Nbs-pFc treated cells compared to medium control in GD-HD inoculated cells may be related to different quantities of the viruses. At 36 hpi, the treatment of Nb6-pFc and Nb53-pFc can induce significant differences in IL-1β, IL-6, IL-8, IL-10, IFN-α, IFN-β, and TNF-α levels on infected PAM, which may be attributed to greater inhibition of PRRSV replication. However, treatment with TAT-Nb53 did not induce significant changes of cytokine production in PAM (Figure 7). It has been reported that energy-independent direct penetration and energy-dependent endocytosis were the two types of entry routes of cell-penetrating peptide mediated entrance [43]. TAT, a cationic cell penetrating peptide with highly positive net charges at physiological pH, can interact with the negatively charged membrane components and phospholipid bilayer to form ‘pores’ for internalization. Therefore, in the present study, at the concentration of 30 μM, TAT-Nb53 directly penetrated into PAM via pores formed in the cell membrane. This mechanism is different from Nbs-pFc used FcγR mediated phagocytosis. Based on these findings, we suggest that Nbs-pFc treatment can induce PAM activation and upregulate cytokine expression, contributing to the inhibition of PRRSV infection of PAM at early stages.

## Discussion

Previously, we reported antiviral nanobody Nb6 against PRRSV Nsp9 [10] and subsequently fused it with TAT to suppress PRRSV replication in MARC-145 cells and PAM [20]. Although the TAT can deliver Nb6 into cells in a time- and dose-dependent manner and dramatically decreases PRRSV replication [20], it showed a random penetration of cells after injection into mice and swine, leading to a very low efficiency for prevention and treatment of PRRS (unpublished data). The tropism of PRRSV is limited to cells of monocyte/macrophage lineage. PAM are firstly infected and mainly replication cells by PRRSV [44]. Fcγ receptors are highly expressed on the cell surface of monocytes and macrophages and play a prominent role in receptor-mediated phagocytosis [22]. In this study, we produced a pig Fcγ and Nb6 fused chimeric nanobody, which can enter PRRSV susceptible cells via FcγR-mediated endocytosis and inhibit PRRSV infection efficiently.

Normally, two heavy chains of antibodies form interchain disulfide bonds in the hinge region. The sequence of pig IgG Fc used here including the hinge region CH2 and CH3. As expected, the apparent molecular weight of Nb-pFc was twice as large after SDS-PAGE run under non-reducing conditions than under reducing conditions (using β-mercaptoethanol) (Figure 1C). Therefore, we hypothesize that Nbs-pFc formed disulphide bonded dimers (Figure 1B). Meanwhile, Nb6-pFc has better antigen binding activity with Nsp9 (Figure 2). In addition, Nb6 expressed in the *Pichia pastoris* system most likely has better protein conformation than that in the prokaryotic system [45].

Currently, it has been reported that there are at least 6 subtypes of porcine IgG [41, 46] with different binding affinities to various types of FcγRs and, thus, different roles in immune regulation [27]. In this work, we used porcine IgG1a since it can bind with FcγRs but not the complement-binding receptors [47, 48]. Cell viability analysis shows that the IgG1a Fc portion fused to Nbs does not induce PAM cytotoxicity at concentrations as high as 50 μM after 24 h of treatment (Figure 4), suggesting that the pig IgG1a subtype is probably safe to be used in future experiments in pigs.

To date, expression of two activating FcγRs (FcγRI, FcγRIII) and one inhibitory FcγR (FcγRIIB) have been identified and characterized on PAM [28–33]. After cross-linking of FcγRI and FcγRIII, tyrosine phosphorylation of the immunoreceptor tyrosine-based activation motifs (ITAM) triggered a series of signal activations and completed the endocytosis process. As expected, Nbs-pFc could be uptaken by PAM, but His-Nb6 without the Fc portion could not (Figure 3A). This result indicates that Fc is a critical factor for the uptake of Nbs-pFc, which first binds with the PAM membranes, then gradually seeps into the cytoplasm (Figure 3D). These results were in agreement with theoretical expectations.

Clover-tagged PRRSV infectious clone and HP-PRRSV strain GD-HD were used to evaluate the inhibition efficiency of Nb6-pFc. Consistent with intracellular expressed Nb6 [10] and TAT-Nb6 [20], two strains of PRRSV were significantly inhibited after treatment with Nb6-pFc (Figures 5 and 6). In contrast with our previous studies [10, 20], Nb53-pFc also decreased PRRSV infection and showed no significant differences with Nb6-pFc at 24 hpi (Figures 5A, C and 6A–C). PAM are equipped with a broad range of receptors and can secrete cytokines by triggering various secondary signaling pathways and produce a variety of cytokines, chemokines, and lipid mediators [25, 26]. Several researchers have demonstrated that FcγRs crossing-linking induces cytokine production [31, 32, 49–51], which are released by FcγR-mediated phagocytosis, depending on the target particle to which the antibody is directed that engages pattern recognition receptors [42]. Therefore, we speculated that antiviral cytokines were induced by FcγRs cross-linking and phagocytosis. Based on the detection of cytokine production in Nbs-pFc treated PAM, we found that anti-inflammatory IL-10 and several pro-inflammatory cytokines (IL-1β, IL-6, IL-8, IFN-β and TNF-α) were significantly upregulated (Figure 7). TAT-Nb53, using the internalization route of direct penetration, did not induce obvious cytokine upregulation (Figure 7) [43]. Several studies have reported that IL-10 can be induced by FcγRs cross-linking [50, 52, 53] and promotes PRRSV infection [53, 54]. Other reports showed that IL-1β, IL-6, IL-12, TNF-α, and IFN-β can also inhibit PRRSV infection [54–56]. In this work, we found that treatment with Nbs-pFc increased the mRNA level of IL-10 significantly in PAM, which was consistent with other investigations [50, 52, 53]. But according to Figure 7, endocytosis of Nbs-pFc induced FcγRs crossing-linking, also revealed other pro-inflammatory pathways, and initiated cytokine production, which exhibited a suppression effect for PRRSV infection.

Figure 5 shows that the purified pig IgG enhanced PRRSV infection, possibly due to the interaction between pig IgG and FcγR that induced PRRSV antibody-dependent enhancement (ADE) [57, 58]. Otherwise, pig IgG is a complex IgG subtype that can strongly bind to the membrane of PAM but cannot be uptaken (data not shown); this may be another reason for the enhancement of PRRSV infection.

During FcR-mediated phagocytosis, particles were first combined into phagosomes, then fused with lysosomes [59, 60]. After 1 h of PRRSV inoculation, Nbs-pFc was added to PAM to inhibit PRRSV replication (Figures 5 and 6). Theoretically, during the early stage of phagocytosis, Nbs-pFc are wrapped by phagosomes and then released or digested by lysosomes, which may explain why the antiviral efficiency of Nb6-pFc at 24 hpi was weak. At 36 hpi, Nb6-pFc presented much stronger antiviral efficiency than Nb53-pFc, indicating that Nb6 interacts with PRRSV Nsp9 and inhibits PRRSV replication. It should be noted that further study is suggested to investigate the release of Nbs-pFc from phagosomes.

PRRSV has a tropism for cells of the monocytic lineage and infects and replicates first in differentiated macrophages, such as porcine alveolar macrophages (PAM) [61]. The results of this study show that Nb6-pFc can suppress PRRSV replication on PAM via FcγR mediated phagocytosis. Thus, to directly target the permissive cells of PRRSV, Nb6-pFc can be delivered by intranasal administration after pigs were infected by PRRSV, which should be evaluated in a future study. In future clinical applications, this Nb6-pFc could be used at any time after virus infection since it targets the virus non-structural protein and blocks virus replication. It could be applied to pigs at a microgram level per head with affordable cost because it could be produced in cell culture systems at a large scale.

## Conclusion

Nanobodies have been widely studied in the diagnosis and treatment of diseases based on their numerous advantages and ability to be modified for specific targets. Currently, PRRS remains a great concern to the swine industry due to the inefficiency of current vaccinations and lack of efficient antiviral strategies. In the present study, an inhibitory nanobody was modified via fusion with a porcine IgG Fc to target PRRSV permissive PAM through FcγR-mediated phagocytosis and dramatically suppress PRRSV replication. This study proposes a novel method to develop porcine IgG Fc fused with targeting nanobodies as the therapeutic agents for PRRS prevention and control.

## Data Availability

All data generated or analyzed during this study are included in the article.
